# Response to stereotactic ablative radiotherapy in a novel orthotopic model of non-small cell lung cancer

**DOI:** 10.18632/oncotarget.22727

**Published:** 2017-11-28

**Authors:** Ayman Oweida, Siham Sabri, Areej Al-Rabea, Mojgan Ebrahimi, Russel Ruo, Richard Fraser, Jan Seuntjens, Bassam Abdulkarim

**Affiliations:** ^1^ Department of Radiation Oncology, University of Colorado Denver, Aurora, Colorado 80045, USA; ^2^ Division of Radiation Oncology, McGill University Health Center, Montreal, Quebec H4A 3J1, Canada; ^3^ Department of Oncology, McGill University Health Center, Montreal, Quebec H4A 3J1, Canada; ^4^ Department of Pathology, McGill University Health Center, Montreal, Quebec H4A 3J1, Canada; ^5^ Medical Physics Unit, McGill University Health Center, Montreal, Quebec H4A 3J1, Canada

**Keywords:** radiation therapy, lung cancer, radioresistance, metastasis

## Abstract

Stereotactic ablative radiotherapy (SABR) is the main treatment for inoperable early-stage non-small cell lung cancer (NSCLC). Despite the widespread use of SABR, the biological determinants of response to SABR remain poorly investigated. We developed an orthotopic NSCLC animal model to study the response to clinically-relevant doses of SABR. Image-guided intra-thoracic injection of NSCLC cells was performed in the right lung of nude rats. A highly conformal dose of 34 Gy was delivered in a single fraction using clinical photon energies. Animals were sacrificed 10–60 days post treatment. Lung tumors were assessed for tumor differentiation, proliferation and invasiveness. An analysis of 770 cancer-related genes was performed on tumor-derived cell lines from treated animals at early and late time points after SABR. The majority of animals receiving SABR demonstrated complete response (67%), while 33% demonstrated local failure. 50% of animals with complete response failed distantly. Analysis of cancer-related genes revealed significant differences between tumors treated with SABR and untreated tumors. SABR significantly modulated expression of genes involved in adhesion, migration and angiogenesis. In particular, interleukin-8 (IL8) which plays a critical role in promoting tumor invasion was found to be secreted at high levels after SABR. *In vitro* invasion assays confirmed SABR-induced invasion and demonstrated induction of IL-8 secretion in multiple NSCLC cell lines. Our findings underscore the importance of developing targeted therapies that can circumvent the pro-invasive effects of SABR in NSCLC.

## INTRODUCTION

Non-small cell lung cancer (NSCLC) represents a highly heterogenous group of tumors with genetic and cellular heterogeneity and diverse pathological features. NSCLC accounts for over 80% of all lung cancer cases and has a 5 year survival rate below 20% [1]. Significant progress has been made in the treatment of early-stage non-surgical NSCLC patients over the last decade. In particular, patients receiving stereotactic ablative radiotherapy (SABR) have local control rates comparable to surgery [2–6]. However, the possibility of offering SABR to operable NSCLC patients remains a contentious issue [7]. Three independent, randomized controlled trials (RCT) in patients with operable stage I non-small cell lung cancer (NSCLC) (STARS, ROSEL, ACOSOG Z4099) were closed early due to slow accrual. While SABR provides excellent local tumor control, emerging data on patterns of failure after SABR show 10% of patients progress locally while 20–30% develop regional and distant metastasis [8–11]. These findings are in contrast to patients who historically would have received conventional fractionated RT where the predominant pattern of failure is local [12]. With increasing dose per fraction, RT deliveries that are highly localized and ablative likely exert distinct biologic effects than conventional doses where damage and repair mechanisms have been well characterized. Available clinical data generally support the application of the standard linear quadratic (LQ) radiobiological model in the range of 1–5 Gy per fraction [13]. Beyond this dose the validity of the standard LQ model to predict response to SABR (in regimens of >5 Gy dose per fraction) is a matter of debate [14]. It is therefore imperative to study the molecular and cellular response to SABR and identify markers of response that can guide patient selection and improve treatment outcomes.

In this work, we characterize tumor response to a clinically-relevant SABR regimen using a novel orthotopic mode of NSCLC. Based on the NSCLC clinical trial by the radiation therapy oncology group (RTOG) 0915 [15] we used a dose of 34 Gy in a single fraction and assessed tumor response at early (10 days), intermediate (30 days) and late (60 days) time points after treatment. We investigated tumor response to SABR pathologically and molecularly. Tumor-derived cell lines were characterized and screened for pro-invasive biomarkers using gene expression profiling of cancer-related genes.

## RESULTS

### Orthotopic induction of NSCLC tumors in rowett nude rats

**Figure 1:**
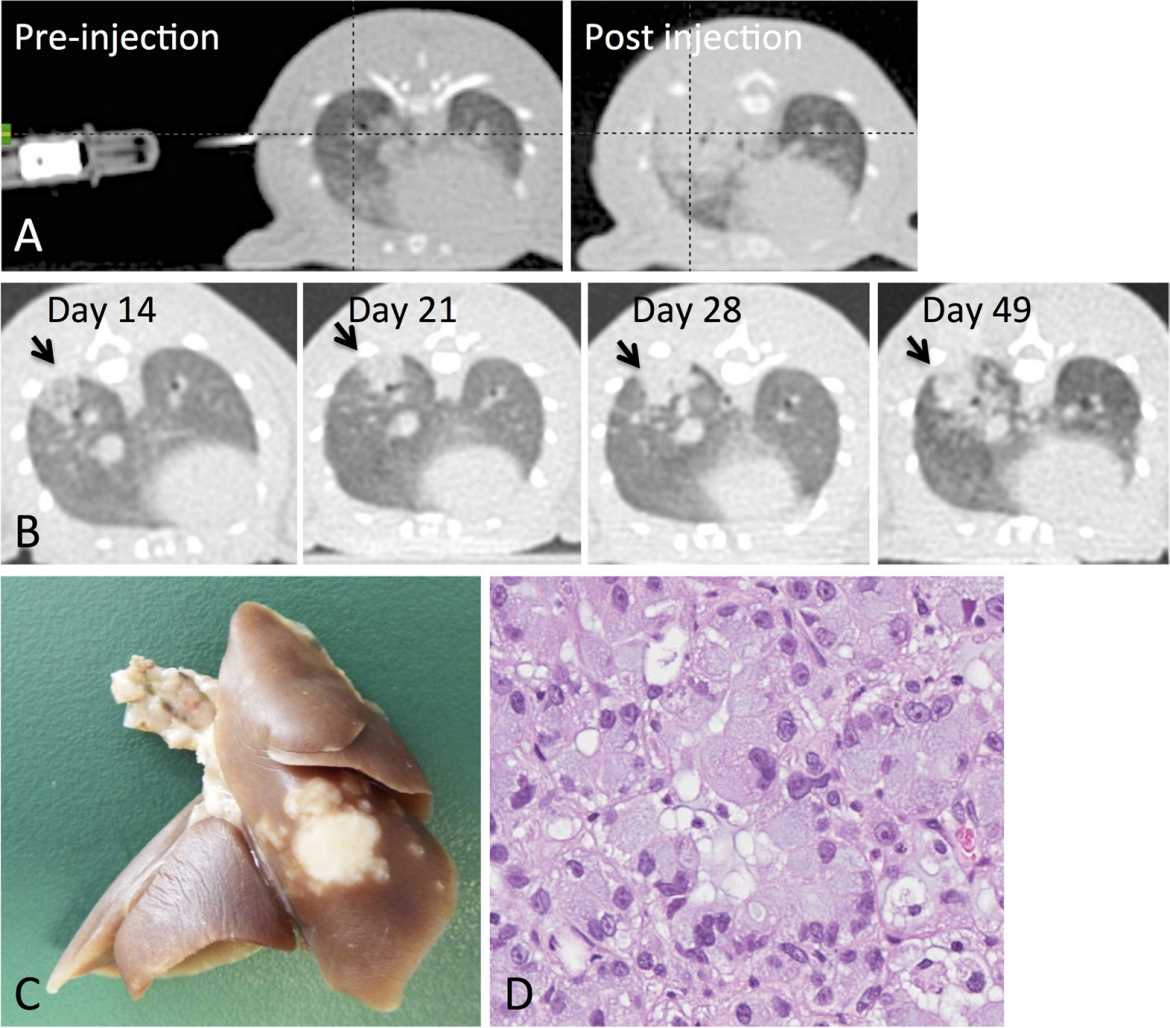
Tumor inoculation and animal follow up (**A**) Animals were pre-positioned and a CT scan was acquired to determine needle position accuracy. A post-injection CT scan was acquired to confirm the success of the injection. (**B**) Longitudinal CT imaging was performed to assess tumor growth and response. (**C**) Representative rat lung showing tumor at the site of injection (right lower lobe). (**D**) H&E shows moderately differentiated adenocarcinoma with mucin production.

Out of 20 rowett nude rats, 20 had successful injections (100% success rate) and 15 developed tumors (75% take-in rate). 4/15 tumor-bearing rats were excluded due to formation of multiple tumor masses in the lung. Only animals with a single primary mass were retained for the study (Figure 1). The remaining 11 animals were randomized to untreated control (*n* = 5) or receiving SABR comprised of 34 Gy in 1 fraction (*n* = 6). No treatment-related toxicities were observed. In addition, no significant difference could be observed in average animal weight between the untreated and 34 Gy groups throughout the course of the experiment.

### Dose distribution and treatment delivery using 6 MV photons

**Figure 2:**
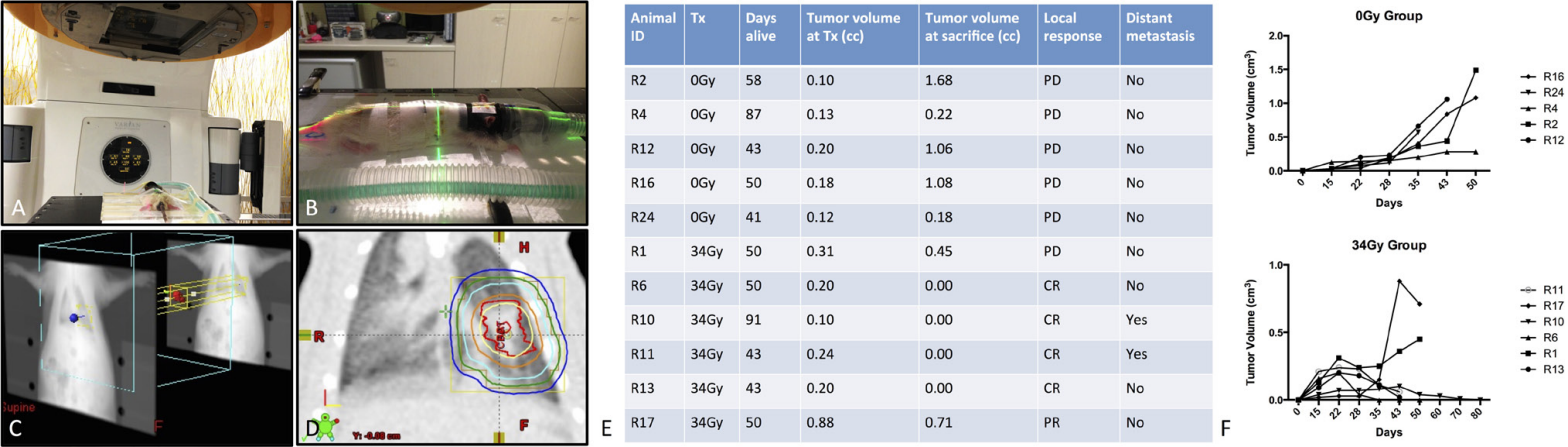
Radiotherapy treatment planning and assessment of tumor growth in rats harboring primary adenocarcinoma of the lung (**A**–**B**) Animal positioning on the Novalis linear accelerator and assignment of beams with multi-leaf collimators to the tumor (**C**–**D**) Treatment planning and target delineation in a rat with a primary NSCLC tumor in the right lung. (**E**) Timeline of each animal's treatment and outcome. (**F**) Individual tumor growth profiles are shown for untreated animals (top) and animals treated with 34 Gy (bottom). Tumor volume was assessed using CT images.

Animals were treated with 3D CRT comprised of anterior and/or posterior beams depending on tumor location (Figure 2A–2D). The distance from the skin surface to the center of the tumor ranged from 1.8 to 2.1 cm. In all cases, the gross tumor volume (GTV) was covered by at least 95% of the total dose (32.3 Gy) with a maximum dose of 102.1% (34.7 Gy). The mean dose to the contralateral lung ranged from 0.3–1.2% of the total dose (average dose: 0.27 Gy; range: 0.10 Gy–0.41 Gy). Dose to the heart ranged from 1.6–16.4% of the total dose (average dose: 2.9 Gy; range 0.54–5.6 Gy).

### Radiological tumor response to SABR in an NSCLC orthotopic animal model

**Figure 3:**
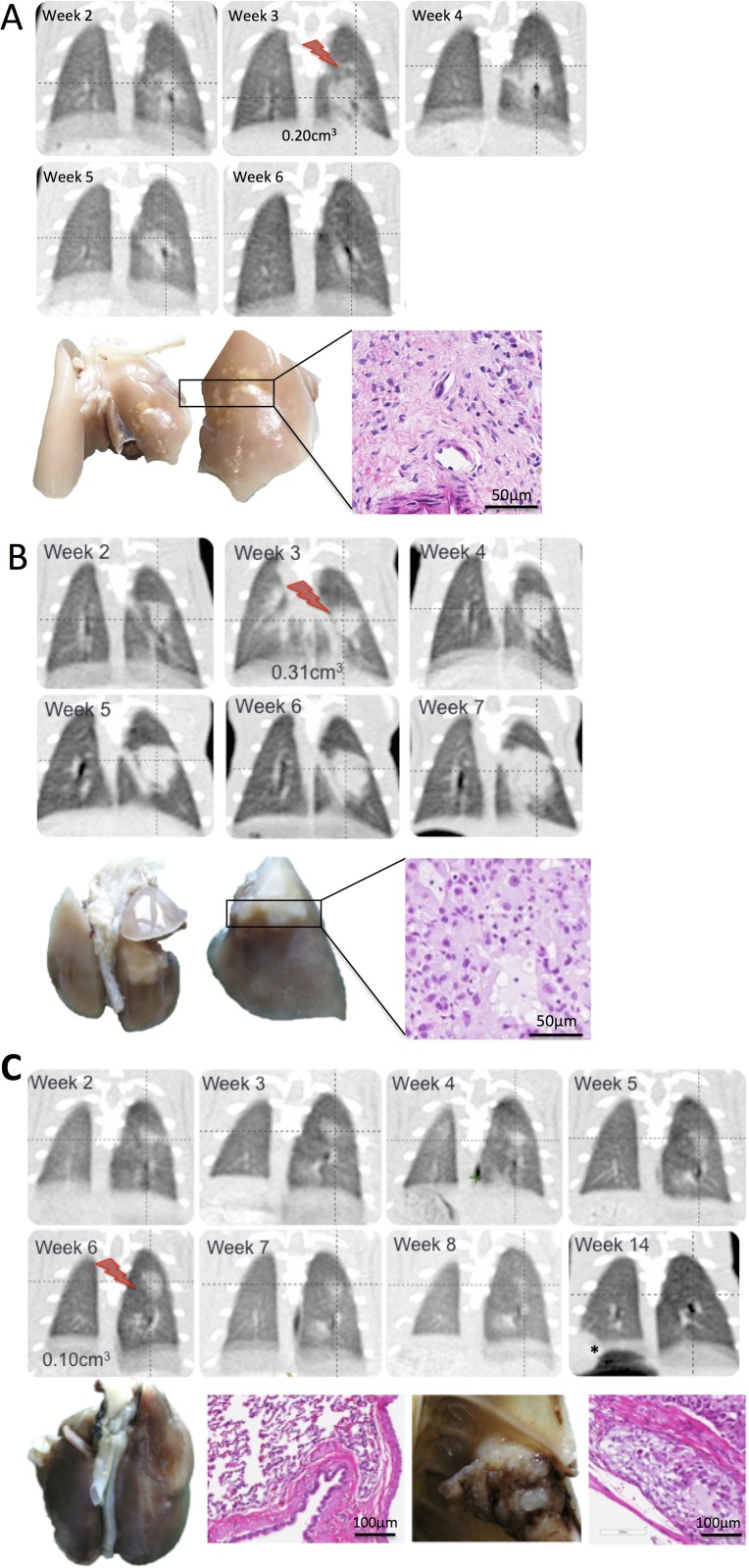
Characterization of tumor response to SABR in an orthotopic model of NSCLC (**A**–**C**) Representative CT images from animals treated with SABR comprised of 34 Gy (red bolt). Gross pathology of the treated lobe is shown as well as H&E staining illustrating characteristic features of tumor response. (A) animal showing disease progression on CT and confirmed pathologically. H&E revealed moderately differentiated adenocarcinoma. (B) Animal showing complete response on CT. Gross pathology revealed discoloration in the treated region which was confirmed on H&E to be comprised of necrotic tissue and immune infiltrates (C) animal showing complete response on CT and later developed distant metastasis.

All untreated control rats had disease progression (PD) and were euthanized based on predefined criteria relating to either excessive tumor burden or to match follow up times in the SABR group (Figure 2E–2F). In the group of animals treated with 34 Gy, 4/6 animals had complete response (CR) with resolution of the tumor within 30 days of treatment; 1 animal had partial response (PR) with tumor shrinking until the time of sacrifice and 1 animal had PD (Figure 2E–2F). Two out of the 4 rats with CR developed distant metastases as observed on CT and confirmed upon pathological assessment with metastasis to bone and skeletal muscle (Figure 3C). Histological assessment of metastatic tumors revealed the presence of poorly differentiated adenocarcinoma (2/2 metastases). This finding is in contrast to all other tumors from treated and untreated animals which exhibited moderately differentiated adenocarcinoma. Osteolytic bone metastasis was evident on CT as a substantial portion of the sixth rib was resorbed and replaced with tumor mass (Figure 3C).

### Pathological tumor response to SABR in an NSCLC orthotopic animal model

Histological assessment of excised lungs revealed the presence of radiation-induced pneumonitis in animals with complete response after SABR (Figure 3A). Two out of the 4 animals with CR exhibited radiation-induced necrosis at the irradiated site (Figure 3A). One animal had disease progression after SABR (Figure 3B). Moderately differentiated mucinous adenocarcinoma was present in all untreated tumors (5/5 animals) as well as treated tumors with PR and PD (2/6 animals).

**Figure 4:**
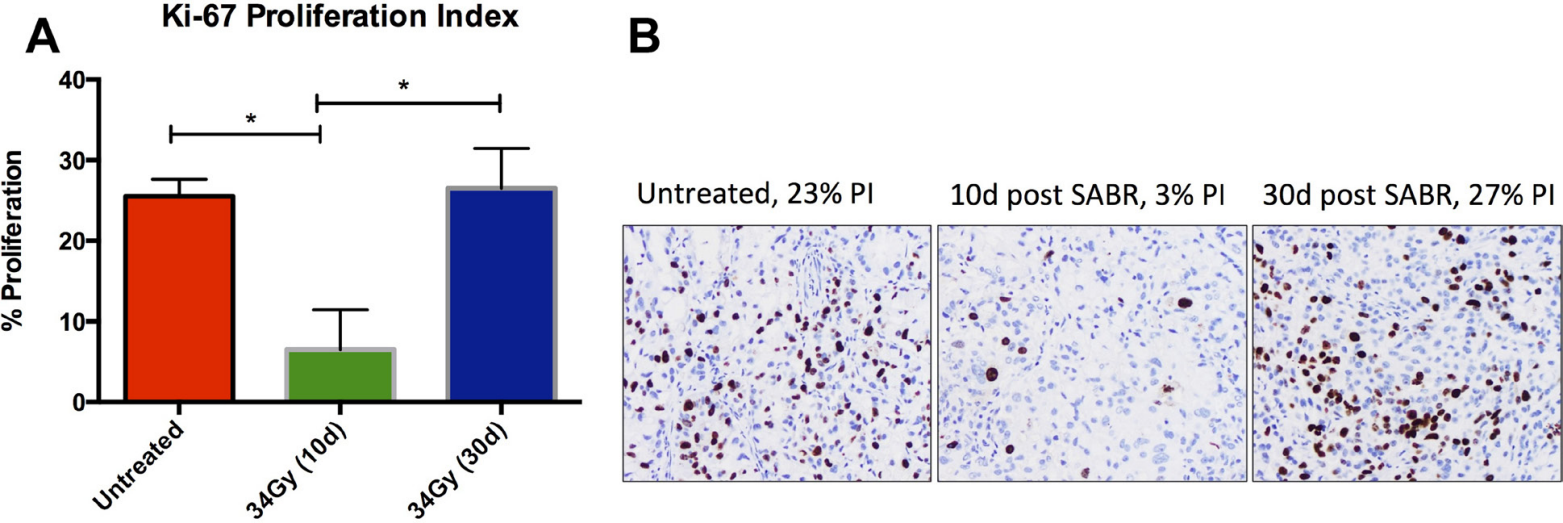
Assessment of cellular proliferation using Ki-67 IHC in tumors excised from untreated tumors and tumors excised at early and late time points after SABR (**A**) Quantitative analysis of the proliferation index of tumors. Statistical significance was determined using ANOVA with *p*-value < 0.05. (**B**) Representative IHC images of Ki-67 and corresponding proliferation index.

Assessment of tumor proliferation is frequently performed using proliferation-associated antigens, including Ki-67 which is expressed throughout the cell cycle of proliferating cells, but not quiescent cells. We investigated the expression of Ki-67 in untreated and SABR-treated animals at early and late time points (Figure 4). Treated animals showed significant reduction in Ki-67 at 10 days compared to untreated tumors. In contrast, Ki-67 score was similar to control in tumors that progressed after SABR and harvested 30–40 days after treatment.

### Assessment of EMT in SABR-treated lung tumors

**Figure 5:**
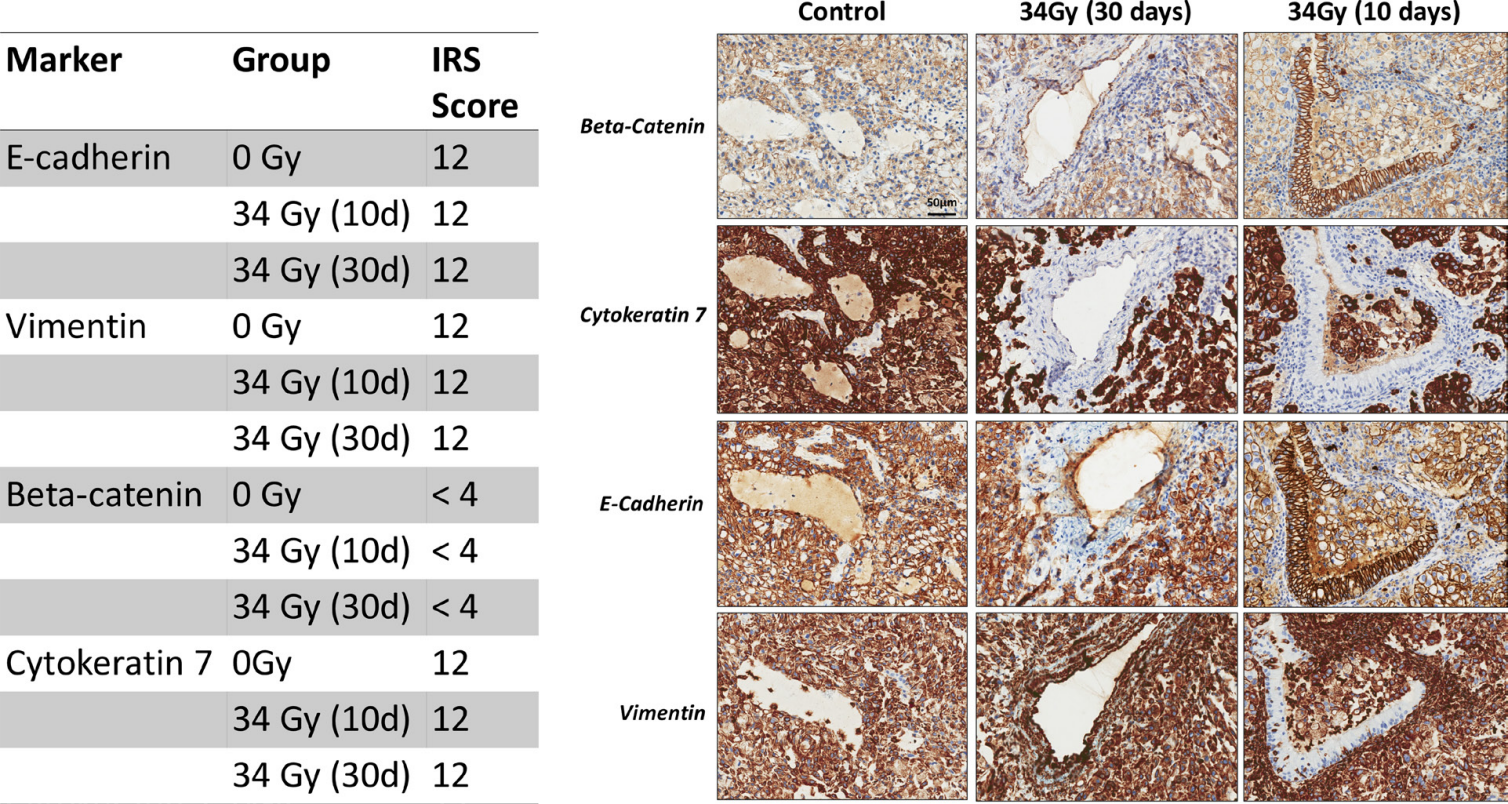
Representative immunohistochemistry images of EMT markers from control tumors and tumors treated with 34 Gy and sacrificed at early ant late time points (10 days and 30 days) Table shows immunoreactivity scores for each stain.

Epithelial-mesenchymal transition (EMT) represents an important mechanism of tumor cell invasion and metastasis. Previous studies on radiation-induced EMT in lung cancer have been controversial and limited to *in vitro* cell assays [16–18]. To our knowledge, there are no studies investigating the expression of EMT markers in SABR-treated tumors *in vivo*. We harvested tumors from rats treated with SABR and assessed the presence of early markers (β-catenin) and late markers of EMT (E-cadherin, vimentin, beta-catenin, cytokeratin 7). In our study, immunohistochemistry of EMT markers did not show differences between untreated tumors and tumors treated with 34 Gy. The expression of E-cadherin, cytokeratin7 and vimentin was high in all analyzed tumors (IRS score = 12). We did not observe a reduction in IRS score of E-cadherin after exposure of tumors to 34 Gy whether at early or late time points by IHC. Gene-expression profiling of cell lines harvested from tumors early and late after SABR revealed a significant decrease in E-cadherin gene expression (CDH1) early after SABR compared to cell lines derived from control tumors as well as late after SABR (Supplementary Figure 1). Since EMT is a dynamic process, it is conceivable that time of sampling can play a role in expression of EMT markers. The expression of β-catenin was membranous in both untreated and treated tumors (Figure 5) and no nuclear or cytoplasmic staining could be observed.

### Genomic and proteomic profiling of SABR-treated tumors

**Figure 6:**
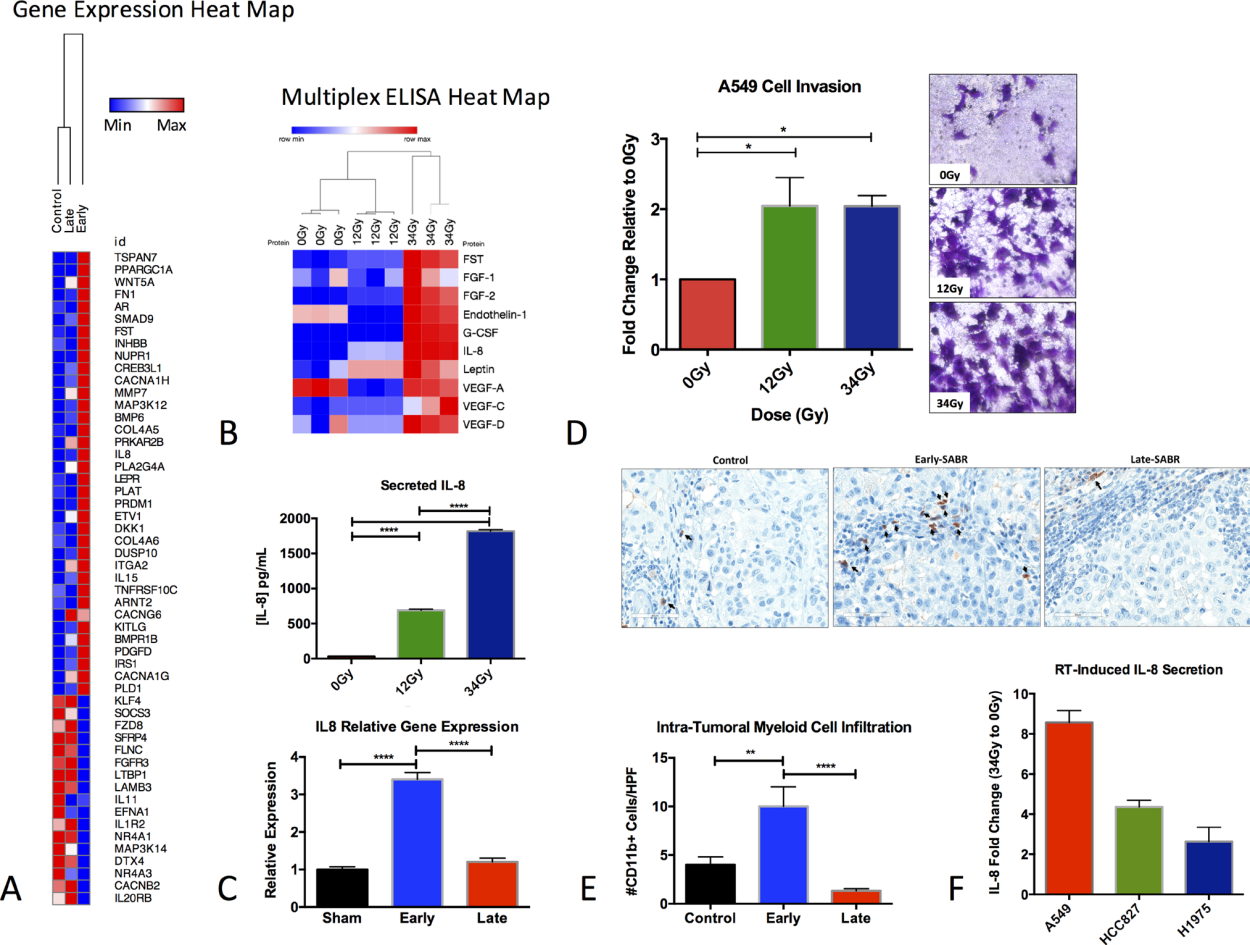
Genomic and proteomic characterization of NSCLC tumors harvested early and late after SABR (**A**) Hierarchical clustering of genes significantly differentially expressed in tumor-derived cell lines from untreated (R16) and SABR-treated animals sacrificed at early (A549R17) and late (A549R1) time points after SABR. Heat map shows genes with greater than 2-fold difference. Adjusted *p* value < 0.05 and Boneferroni correction applied. (**B**) Hierarchical clustering of secreted proteins analyzed with multiplex ELISA from A549 conditioned media 72 hours after treatment with 12 Gy or 34 Gy. (**C**) Quantitative analysis of relative gene expression and ELISA for IL-8. (**D**) Quantitative analysis of matrigel invasion assay of A549 cells treated with 12 Gy and 34 Gy compared to control 0 Gy. (**E**) IHC analysis of CD11b+ myeloid cell infiltration from control tumors and tumors harvested at early and late time points after SABR. Representative images acquired at 400× magnification are shown. (**F**) Analysis of IL-8 by ELISA in conditioned media from different NSCLC cell lines exposed to 34 Gy. Data is presented as fold change relative to 0 Gy. Bars represent SEM from 2–4 independent experiments.

Two cell lines were derived from SABR-treated animals: one cell line was harvested from a primary tumor, 30 days after SABR (A549R1) and one cell line was harvested from a primary tumor, 10 days after SABR (A549R17). In addition, one cell line was harvested from a primary untreated lung tumor (A549R16) (Supplementary Table 1). To discern the genomic profile of the tumor-derived cell lines, we performed gene-expression profiling using the NanoString nCounter for a specific number of cancer-related genes (770 genes). The nCounter detects total mRNA counts through hybridization with fluorescently labeled bar coded probes to the mRNAs of interest followed by scanning and counting to quantify expression [19]. We observed significant differences in gene-expression between the two SABR-treated tumor-derived cell lines and the control cell line (Figure 6A). Our analysis showed 165 significantly differentially expressed (DE) genes in A549R1 (Late) compared to the untreated A549R16 tumor-derived cell line (adjusted *p* value < 0.05, Bonferroni correction) and 270 significantly DE genes in A549R17 (early) compared to A549R16 (adjusted *p* value < 0.05, Bonferroni correction). Gene ontology (GO) analysis revealed a set of genes related to adhesion and migration that are upregulated in A549R17 compared to the other cell lines, including follistatin (FST), interleukin 8 (IL-8), fibronectin 1 (FN1), matrix metalloproteinase 7 (MMP7) and tetraspanin 7 (TSPAN7) (Supplementary Table 2). Multiplex ELISA analysis of secreted factors confirmed significantly increased levels of IL-8 in conditioned media from A549 cells exposed to 12 Gy and 34 Gy doses of radiation compared to non-irradiated cells (Figure 6B, 6C). To determine whether ablative radiation has an effect on A549 cell invasion, we performed matrigel invasion assays on A549 cells irradiated with 12 Gy, 34 Gy or control. Our results demonstrate a significant increase in invasion of A549 cells after exposure to 12 Gy and 34 Gy (Figure 6D), consistent with our previous findings [20]. Since IL-8 is a potent chemoattractant for macrophages and neutrophils, we assessed intratumoral infiltration of myeloid cells (CD11b+ staining) from tumors harvested early after SABR, late after SABR or control. We observed a significant increase in myeloid cells early after SABR treatment which normalized at the later time point (Figure 6E). To confirm that SABR-induced IL-8 secretion is not cell-type specific, we additionally assessed IL-8 secretion in conditioned media from other irradiated lung cancer cell lines with genetic background (H1975 and HCC827 cells). A dose of 34Gy induced a 4.4-fold increase in IL-8 secretion in HCC827 cells, 2.6-fold increase in H1975 cells and 8.6-fold increase in A549 cells (Figure 6F). Collectively, our results reveal a role for SABR in promoting secretion of IL-8 which can contribute to increased invasion and metastasis.

## DISCUSSION

Technological advances in radiation therapy have created a paradigm shift in the treatment of early stage lung cancer. The shift from conventionally fractionated radiotherapy to SABR has resulted in significant gains in tumor locoregional control, patient quality of life and overall survival for early-stage inoperable NSCLC patients [21, 22]. However, the experience with SABR remains young, especially when compared to surgery for operable early-stage NSCLC patients. Furthermore, radiobiological models utilized in conventional fractionation schemes may not apply to SABR regimens [22]. It is therefore imperative to develop pre-clinical animal models of lung cancer in order to elucidate molecular mechanisms of radioresistance and tumor response after SABR.

Previous studies on animal models of NSCLC for radiotherapy have focused on proof of principle of technology involving kV x-ray (160–250 kVp) energies which are considerably lower than MV photon energies used in the clinic [23–25]. Such studies showed the feasibility of delivering conformal radiation to normal lung tissue lacking tumor. In the first study of its kind, Saha *et al.* demonstrated successful targeting of a rat lung tumor using image-guided SABR with 250 kV x-rays [26]. The study showed the feasibility of inducing and targeting a primary tumor in rat lung, but the choice of dose regimen (60 Gy in 3 fractions) did not translate to therapeutic efficacy and only results from 1 animal per group were reported. To our knowledge, our study is the first to show delivery of clinically relevant SABR using MV photon energies to tumor-bearing animals and demonstrate therapeutic efficacy similar to that seen in the clinic. This model will allow further exploration of the genomic and cellular response of NSCLC tumors to SABR doses.

Epithelial to mesenchymal transition is an important process in the development of metastasis [27]. EMT involves the phenotypic transformation of epithelial cells to mesenchymal-like cells. In most epithelial cancers, E-cadherin mediated cell-cell adhesion is lost with increased tumor progression and metastatic spread. In contrast, expression of the intermediate filament, vimentin is characteristic of mesenchymal cells [28]. Several studies have shown that RT can induce EMT and increase invasion *in vitro* in A549 lung adenocarcinoma cells [17, 18, 29]. In contrast, other studies could not detect EMT induction after radiation [16, 30]. An important aspect of our study was to investigate whether a SABR dose of radiation can stimulate EMT *in vivo* when tumors are in their natural microenvironment. We harvested tumors from rats treated with SABR and assessed the presence of early markers of EMT (β-catenin) and late markers of EMT (E-cadherin, vimentin, cytokeratin). We could not detect changes in the EMT markers, E-cadherin, vimentin, cytokeratin or β-catenin at early or late time points after SABR by IHC. It is pertinent however to recognize that EMT is a transient and reversible process and our choice of time points may have missed this process.

The absence of an EMT phenotype after SABR lead us to perform gene-expression profiling using Nanostring on cell lines derived from the same tumors. Cancer-related gene-expression profiling of tumor-derived cell lines from SABR-treated animals showed significant differences in gene-expression compared to untreated animals. In particular, at early time point after SABR (10 days) important genes involved in metastasis were modulated including, FN1, FST, MMP7 and IL-8. Consistent with gene-expression data, cytokine profiling revealed a consistent trend in the upregulation of IL8 after SABR in a dose-dependent manner. IL8 is an important chemokine and key regulator of myeloid cell recruitment including, neutrophils and macrophages. Upon binding to its G protein-coupled receptors, CXCR1 and CXCR2, IL8 can activate a cascade of signaling pathways involved in cell proliferation, survival, invasion and angiogenesis [31]. Our observation of increased myeloid cell infiltration in NSCLC tumors after exposure to 34 Gy is consistent with the observed increase in IL-8 levels. In addition to its direct effects on migration and invasion, IL8 can induce tumor-associated macrophages to secrete pro-invasive and angiogenic growth factors that can further stimulate tumor cell invasion and metastasis [31]. Interestingly, analysis of IL8 in lung adenocarcinoma patients revealed a significant positive correlation between IL8 expression, KRAS mutation status, disease-free survival and overall survival [32]. Future animal experiments in our laboratory are aimed at exploring the combination of SABR with targeted therapies blocking IL8 in order to counteract the pro-invasive effects post-SABR.

One of the limitations of our study is the small number of animals per time point in the group receiving SABR. Future studies involving cross-sectional assessment of tumors at multiple time points after SABR will provide critical insight into mechanisms of failure and guide the rational design of targeted therapies in combination with SABR.

In conclusion, we developed a pre-clinical animal model to assess tumor response to SABR. The rat orthotopic model of human NSCLC adenocarcinoma can effectively mimic the heterogeneity of lung cancer biology and response to treatment observed in human tumors. Further validation of genes modulated in SABR-treated animals that develop metastasis is an important next step. Our genomic and proteomic analysis of SABR-treated tumors provides a framework for development of targeted therapies that can inhibit the pro-invasive response post-SABR.

## MATERIALS AND METHODS

### Cell lines

Human lung adenocarcinoma cells A549, H1975 and HCC827 were obtained from the American type culture collection (ATCC, VA). Cells were cultured at 37°C and 5% CO2 in RPMI 1640 media except for A549, which was cultured in DMEM F/12 media. Media was supplemented with 10% fetal bovine serum (FBS) and 1% penicillin/streptomycin. Cell irradiation was performed using a Faxitron X-Ray machine (Faxitron X-ray Corporation, IL). X-ray tube voltage was set to 160 kVp, current of 6.3 mA and dose rate of 1450 R/min.

### Tumor implantation

Human lung adenocarcinoma A549 cells were used to induce a single primary tumor in the right lung of nude rats. 10^6^ cells were mixed with matrigel (1:1) in a final volume of 50 ul. Cells were injected in the intercostal space corresponding to the lower lobe of the right lung using a 28G1/2 needle. A landmark was placed at the site of injection and CT imaging (Philips, Brilliance CT Big Bore) was performed to confirm injection site accuracy. A needle guard was used to limit the depth to 0.8 cm beyond the entry point. While maintaining the animal in the same position, a post-injection CT was acquired to confirm the injection was successfully delivered to the lung. Only animals with a successful injection from the first attempt were kept for the study. All procedures were performed with the approval of the McGill University Animal Care Committee.

### Imaging and tumor growth

For effects on tumor growth and delay, CT imaging was performed weekly. Tumor volume was measured using clinical treatment planning software (Varian, Eclipse™V11). Follow-up time ranged from 10–60 days after treatment. Tumor response was assessed by weekly CT imaging after treatment consistent with the RECIST criteria [33]. Complete response (CR) was defined as the disappearance of the target mass. Partial response (PR) was defined as a reduction of at least 30% in the tumor volume. Progressive disease (PD) was defined as at least a 20% volume increase in the treated mass. Stable disease was assumed if neither PR or PD could be observed.

### Treatment planning and delivery

Animals underwent CT simulation immediately prior to radiation delivery. Treatment planning was performed using the Eclipse ™treatment planning software. The AAA heterogeneity correction was applied. Radiation was delivered using a Varian Novalis TX linear accelerator with 6 MV photons and multi-leaf collimators (MLC) customized to the gross tumor volume (GTV). The clinical target volume (CTV) was defined as coinciding with the GTV and a planning target volume (PTV) with a 0.2 cm margin was added to the CTV.

### Tissue processing and tumor histology

Under deep anesthesia, animals were perfused with 250 mL PBS and fixed with 10% formalin. Prior to lung excision, the trachea was infused with 3 mL of formalin through a needle-size incision and clipped below the incision. Lungs were excised along with brain, liver, spleen and mediastinal lymph nodes and fixed in 10% formalin for 48 h. All experimental animals were assessed pathologically by a certified pathologist for the presence of regional and distant metastasis. When the presence of tumor was suspected on gross pathology, sections were taken for microscopic assessment of H&E slides. Specimens were processed and stained with H&E at immunohistochemistry core facility. Tumor samples were analyzed by 2 independent pathologists for tumor type, histologic grade as well as histological changes including the presence of tumor necrosis, apoptosis, mitotic activity and fibrosis.

### Tumor immunohistochemistry

All the histopathological sections were stained on the Dako Omnis platform with Envision Flex Dab kit. Antibodies to vimentin (GA63061), E-cadherin (GA05961), Ki-67 (GA62661), beta-catenin (IR70261), cytokeratin 7 (A61961) and CD11b (WT.5) were added to individual sections. Slides were dehydrated and mounted with cover slips. Positive cells were identified visually. In the case of Ki-67, the percentage of positive cells was calculated by dividing the number of positive cells by the total number of cells in each high-power field (40× magnification). For CD11b infiltration, the number of positive stained cells per high power field (HPF) was determined. A total of 10 random fields was analyzed per sample from at least 3 specimens. For the remaining IHC stains, the immunoreactivity scoring system (IRS) was used as proposed by Remmele and Stegner (REF) with slight modification as follows: IRS = the product of staining intensity (SI) and percent positive staining (PP). SI was quantified as 0, negative; 1, weak; 2, moderate; and 3, strong. PP was determined as 0, negative; 1, 1–10% positive cells; 2, 11–50% positive cells; 3, 51–80% positive cells; 4, 81–100% positive cells. An IRS score of greater or equal to 4 was considered as a positive staining result [34]. A total of 10 fields were analyzed.

### Tumor-derived cell lines and gene expression profiling

Cell lines were derived from SABR-treated animals. One cell line was harvested from a primary tumor, 10 days after SABR (A549R17) and one cell line was harvested from a primary tumor 30 days after SABR (A549R1). In addition, one cell line was harvested from a primary untreated lung tumor (A549R16). Tumor-derived cell lines were cultured for 3–4 passages and washed 5 times per passage to ensure removal of stromal components before performing RNA extraction. RNA extraction was performed using Trizol (Sigma-Aldrich) and purified using Qiagen RNeasy columns (Qiagen Sciences, Maryland) according to manufacturer's instructions. The RNA was quantified using NanoDrop 1000 spectrophotometer and its integrity evaluated using a Bioanalyzer 2100 (Agilent) according to manufacturer's protocols. Extracted RNA was stored at –80°C until analysis. Expression of 770 cancer-related genes was measured using the NanoString nCounter mRNA expression assay kit (NanoString Technologies, Seattle, WA). The nCounter detects total mRNA counts through hybridization with fluorescently labeled bar coded probes to the mRNAs of interest followed by scanning and counting to quantify expression [19]. Relative expression was determined by normalizing the number of reads in SABR-treated groups relative to control.

### ELISA analysis

We quantified 17 angiogenesis/growth factor biomarkers (angiogenesis and growth factor array, Eve Technologies Corp, Calgary, AB) using multiplex immunoassay analyzed with a BioPlex 200 system (Bio-Rad Laboratories, Inc., Hercules, CA, USA). The 17-plex consisted of Angiopoietin-2, BMP-9, EGF, Endoglin, Endothelin-1, FGF-1, FGF-2, Follistatin, G-CSF, HB-EGF, HGF, IL- 8, Leptin, PLGF, VEGF-A, VEGF-C, VEGF-D. For this set of experiments, conditioned media from untreated, 12 Gy irradiated and 34 Gy irradiated A549 cells was obtained by incubating cells for 24 hours with serum-starved media 6 days after exposure to RT. We further validated IL-8 levels in additional cell lines using the IL-8 Quantikine ELISA kit (D8000C, R&D Systems, MN, USA.

### Statistical analysis

Two-way analysis of variance (ANOVA) was used to compare IHC scores between groups. A *p*-value less than 0.05 was considered significant. Statistical analysis was performed in Prism software. For gene-expression data, NanoStriDE was used for normalization and differential expression analysis as previously described [35].

## SUPPLEMENTARY MATERIALS FIGURES AND TABLES



## Abbreviations

SABR: stereotactic radiotherapy
NSCLC: non-small cell lung cancer
EMT: epithelial to mesenchymal transition
Gy: Gray.
