# Supplementary material for: Response to stereotactic ablative radiotherapy in a novel orthotopic model of non-small cell lung cancer
Source: Oncotarget. 2017 Nov 28;9(2):1630–40. doi: 10.18632/oncotarget.22727 (PMC5788587; doi:10.18632/oncotarget.22727)
Supplement: Supplementary file 1 [file oncotarget-09-1630-s001.pdf]

Response to stereotactic ablative radiotherapy in a novel orthotopic model of non-small cell lung cancer

SUPPLEMENTARY MATERIALS

Supplementary Table 1: Characteristics of tumor-derived A549 cell lines

Cell line ID	Treatment	Endpoint	Site of cell line
A549R16	None	Control	Primary
A549R1	34 Gy	30 days	Primary
A549R10	34 Gy	10 days	Primary

Supplementary Table 2: Relative expression of genes involved in adhesion/migration in A549 cells derived from sham-treated tumors or tumors harvested early and late after SABR

	Relative gene expression (St.Dev.)		
	Sham	Early	Late
PPARGC1A	1.00 (0.40)	17.4 (4.80)	0.90 (0.30)
TSPAN7	1.00 (0.10)	17.1 (1.20)	1.30 (0.20)
FN1	1.00 (0.01)	9.90 (0.60)	1.50 (0.10)
FST	1.00 (0.03)	5.50 (0.20)	0.60 (0.01)
IL-8	1.00 (0.10)	3.40 (0.20)	1.20 (0.10)
BMP6	1.00 (0.10)	3.40 (0.30)	1.20 (0.20)
COL4A5	1.00 (0.10)	3.10 (0.20)	0.80 (0.10)
COL4A6	1.00 (0.02)	2.30 (0.10)	0.60 (0.02)
IL20RB	1.00 (0.10)	0.10 (0.01)	1.60 (0.20)

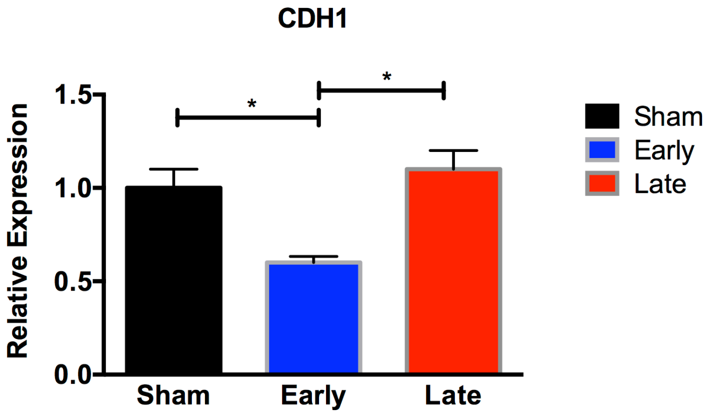

Supplementary Figure 1: Relative gene expression of CDH1 (E-cadherin) in A549 cells harvested from sham-treated tumors and tumors harvested early and late after SABR.
